# A machine learning-based approach for sentiment analysis on distance learning from Arabic Tweets

**DOI:** 10.7717/peerj-cs.1047

**Published:** 2022-07-26

**Authors:** Jameel Almalki

**Affiliations:** Department of Computer Science, College of Computer in Al-Leith, Umm Al-Qura University, Makkah, Saudi Arabia

**Keywords:** Sentiment analysis, Social media, E-Learning, Twitter, Apache Spark, Arabic language

## Abstract

Social media platforms such as Twitter, YouTube, Instagram and Facebook are leading sources of large datasets nowadays. Twitter’s data is one of the most reliable due to its privacy policy. Tweets have been used for sentiment analysis and to identify meaningful information within the dataset. Our study focused on the distance learning domain in Saudi Arabia by analyzing Arabic tweets about distance learning. This work proposes a model for analyzing people’s feedback using a Twitter dataset in the distance learning domain. The proposed model is based on the Apache Spark product to manage the large dataset. The proposed model uses the Twitter API to get the tweets as raw data. These tweets were stored in the Apache Spark server. A regex-based technique for preprocessing removed retweets, links, hashtags, English words and numbers, usernames, and emojis from the dataset. After that, a Logistic-based Regression model was trained on the pre-processed data. This Logistic Regression model, from the field of machine learning, was used to predict the sentiment inside the tweets. Finally, a Flask application was built for sentiment analysis of the Arabic tweets. The proposed model gives better results when compared to various applied techniques. The proposed model is evaluated on test data to calculate Accuracy, F1 Score, Precision, and Recall, obtaining scores of 91%, 90%, 90%, and 89%, respectively.

## Introduction

Nowadays, the role of the Internet has changed with people using social media as an alternate to traditional news media. Blogs, Twitter, Facebook, and various other social media platforms (Appel, Grewal & Hadi, 2020) are used to get instant information about anything in the world. Many people use social media only for the latest news and other information about the latest trends in any field of life. Sentiment analysis of opinion on distance learning is an approach to determine whether actual outcomes match desired success. A usable source of media is Twitter, which is very reliable due to its worldwide usage and enhanced security of people’s data according to the user privileges. It provides factual information and always relies on unique details. Many educational institutes use social media to share their latest news and knowledge (Schneider & Council, 2021). The Twitter platform (Kapoor et al., 2018) is commonly used for this purpose.

In addition to its use as a news source, educational systems use Twitter for analysis of feedback on the quality of education and to check their students’ trust in the educational system. In particular, people use Twitter to discuss distance learning education techniques. In Saudi Arabia, many institutes facilitate distance learning (Yu & Qi, 2018) education and would like to know how people feel about this mode of delivery. The COVID-19 Twitter dataset plays an essential role in understanding and learning about actual thinking about distance learning. It is also used to check the various studies discussing distance learning.

Distance learning (Khalil et al., 2020) is the approach in which the student does not always appear physically in the classrooms to attend the classes. In the past they were facilitated *via* paperwork resources. Nowadays, it has been transferred online where students attend courses *via* online tools like Skype, Zoom, Microsoft Teams, and Google Hangouts (Adwan et al., 2020).

As mentioned above, due to physical distancing requirements imposed by the COVID-19 pandemic, almost all institutes have been using online resources. Distance learning has enable students to continue with their education while at home and has potentially saved time with the need to travel to the classroom removed. Distance learning allows students to get course content easily and, if they are unable to attend the class physically, allows them to attend all classes online. The various benefits of using distance learning, or e-learning (Matar, 2017), systems were realized during the Covid-19 pandemic when all the institutes transferred to e-learning systems.

E-Learning has some drawbacks, such as the quality of education being disturbed. Individual students may face different qualities of internet connection and may encounter problems that go unresolved due to non-availability of technical staff. Our research focuses on these points where there are drawbacks and benefits for the e-learning education system. The distance learning Twitter dataset can identify whether e-learning is being used or not. Tweets can be checked for whether people are thinking positively or negatively and can also identify neutral opinions about distance learning in Saudi Arabia. We can identify whether distance learning (Khan & Alotaibi, 2020) is a better approach or not during the Covid-19 pandemic. Based on the analysis of the sentiment found in tweets, we can suggest decisions and rules for effective e-learning.

Distance learning (Arambepola, 2020) plays a role in facilitating learning in Saudi Arabia. People learning *via* this educational methodology are using social media to discus the distance learning concept. This social media discussion can be analysed to identify people’s sentiment towards distance learning in Saudi Arabia. Our proposed method targets distance learning commentary in Arabic tweets to analyze whether people think positively, neutrally, or negatively. Various techniques are applied to analyze distance learning on Arabic tweets (Georgescu & Bogoslov, 2019). We deal with the problem of analyzing the Twitter dataset for distance learning using machine learning techniques. Apache Spark (Hasan et al., 2019) was used to serve, manage and analyse the dataset. https://spark.apache.org/ is a data processing framework that can process tasks quickly and easily in very large databases. It can execute its operations across distributed computers, distributed databases and other relevant tools. These qualities of the spark are a key to the words of machine learning and big data, which requires the large computing power to crunch the large datasets. Spark also takes some of the programming burdens of these tasks off the shoulders of developers with an easy-to-use API that abstracts away much of the grunt work of distributed computing and big data processing.

This article presents the complete flow of the proposed model in this introduction, the literature review in the next section, and, subsequently, the proposed methodology. Then, results from the model are discussed and conclusions presented.

This research mainly contributes to the domain of sentiment analysis, as we propose a model for analyzing people’s feedback using a Twitter dataset in the distance learning domain. The accuracy of the model is quite satisfactory and the proposed research could be helpful for the decision-makers while making the policies for distance learning.

## Literature Review

In this section, various techniques for sentiment analysis using the Twitter dataset in the distance learning domain has been discussed. The goal is to develop a solution for the distance learning sentiments analysis of Arabic tweets. Bhaumik & Yadav (2021) proposed a technique for sentiment analysis on the Twitter dataset in different domains like graduates and officials. In this technique, tweets were obtained *via* the Twitter API. Then, they preprocess the tweets, pass these tweets to the Valence Aware Dictionary for Sentiment Reasoning (VADER) model to get the sentiment analysis for the given tweets. Sentiment analysis for distance learning tweets is discussed in this technique. Kharde & Sonawane (2016) proposed a method to analyze the Twitter dataset for the distance learning domain. The tweets were accumulated using the Twitter API and the compiled dataset was analyzed. They used machine learning techniques to identify sentiment in the tweets, using a classifier to classify positive and negative sentiment.

A novel approach implemented in this article where they used the twitter distance learning tweets for the model. They scrapped the Twitter dataset using the Twitter API. Then, they applied preprocessing techniques to remove outliers like links, usernames, and special characters. They applied machine learning techniques (Krouska, Troussas & Virvou, 2016) to identify sentiment in tweets for different datasets including distance learning tweets. Deep learning techniques (Zhang, Wang & Liu, 2018) were applied to this model to get the best results for the sentiment of positive and negative for the overall dataset. They also discussed the deep learning methodology for the sentiment analysis of the Twitter dataset. Authors also discussed opinion mining and decision mining techniques for user review. A deep learning approach is best for the sentiment analysis, which is implemented in this research study.

Althagafi et al. (2021) depicted that tweeting is a decent method of identifying popular assessment about web-based learning, as the stage is boundless in Saudi Arabia. Estimation investigation is the work of perceiving idealistic and negative perspectives, sentiments, and assessments. Slant examination can be directed at a few levels such as levels of the report, sentence, and subject. In this exploration, the authors are keen on the sentence level assessment examination of Arabic tweets in order to survey the tweet extremity; regardless of whether it is positive, negative, or impartial. We are keen on assessment grouping in the Arabic language on a sentence-by-sentence basis in which the point is to characterize tweets about web-based learning in Saudi Arabia to decide individuals’ conclusions identified with this subject and group the tweets to positive, negative, or nonpartisan. Our examination was focused on twitter’s assessment mining and slant examination about web-based learning picking up during the COVID-19 pandemic, which bifurcates tweets dependent on three classes such as positive, negative, and nonpartisan. We identified that most tweets were communicating a nonpartisan slant, which may have occurred since a significant number of tweets featured statements that did not communicate either negative or positive emotions.

Numerous understudies (Osmanoglu et al., 2020) of the open colleges are utilized, previously working people. A large number of them barely figure out how to consider and, hence, search for learning materials that they can get to whenever. In this way, advanced learning materials become much more significant for distance students than at any other time. Distance schooling can be characterized as “any learning exercises inside formal, casual, and non-formal areas that are worked with data and correspondence advances to decrease distance, both actually and mentally, and to expand intelligence and correspondence among students, sources and facilitators of learning” as well as the topic of distant learning instruction has changed significantly after the 2000s when a change in perspective saw due to limit increment got from ICT and online arranged advancements. In this examination, scientists will break down criticisms assembled from the e-Campus framework by utilizing AI methods. The informational index assets utilized in this examination were criticisms of the distance students. As needs are, in distance schooling frameworks, where learning happens, such AI based methods can be utilized and it is also used to get experiences how students in these frameworks focus. In any case, it ought to be noticed that the achievement of examinations has gone from 42% to 85%. Probably the greatest limitation of supposition examination through translation is the non-recognition of the language rules. Because of comparable reasons, the exactness stays wide.

The purpose of this research in Aljameel, Alabbad & Alzahrani (2021) was to look into Saudi Arabian residents’ awareness of the COVID-19 pandemic and their attitudes toward epidemic control. The targets were fine-tuned by gathering and compiling data from Saudi Arabia’s five regions: north, south, east, west, and central. Because Twitter is such a popular platform in Saudi Arabia, tweets were the most important source of information for this inquiry. The target of this examination was to assemble and set up a dataset that portrays an individual’s care, and a short time later to use the dataset to asses every space in Saudi Arabia. The conjecture of care will help clinical regions and the public authority to uncover issues and make the right decisions to control the pandemic. An AI model was developed to arrange and predict an individual’s thoughts regarding the judicious strategies based on tweets in the five regions of the Kingdom of Saudi Arabia. Finally, using the constructed model, individual knowledge is anticipated in Saudi Arabia’s five critical places while taking into account the degree of caring among these areas.

In recent years, the disciplines of machine learning and Web technology have seen tremendous advancements. This resulted in a continuous and rapid increase in the sharing of opinions and experiences on services and goods through the Internet in many sectors. As a result, for analytical investigations, a torrential flow of online data is available. Sentiment Analysis (SA) is a subtask of Natural Language Processing (NLP) that tries to evaluate large amounts of data to discover people’s thoughts and feelings. This subject has piqued the interest of both the public and business sectors, resulting in a slew of problems, particularly those relating to the Arabic language. The purpose of this study (Nassif et al., 2020) is to conduct a thorough assessment from 2000 to June 2020 in order to assess the level of deep learning for Arabic NLP (ANLP) activities in Arabic Subjective Sentiment Analysis (ASSA), as well as to highlight challenges and research directions in this field. A wide number of researches were analyzed to examine deep learning algorithms utilized in subjective sentiment analysis for the Arabic language. The most often utilized ASSA techniques were CNN and RNN (LSTM) models, according to our findings. The findings of this study indicate that further work is needed to adopt updated deep learning approaches for Arabic sentiment analysis systems.

This research proposed a novel approach for sentiment analysis of distance learning using Arabic tweets. In our proposed technique, API model is designed which scrapes the tweets using the Twitter API. The dataset obtained is stored using Apache Spark (Elzayady, Badran & Salama, 2018). Scrapped data is preprocessed to remove unnecessary content, for example links, usernames, and special characters. This data is passed to the training model which trains for the sentiment. A Flask application provides an interface to the trained model to allow the sentiments of new tweets to be identified as positive, negative, and neutral.

## Proposed Methodology

**Figure 1:**
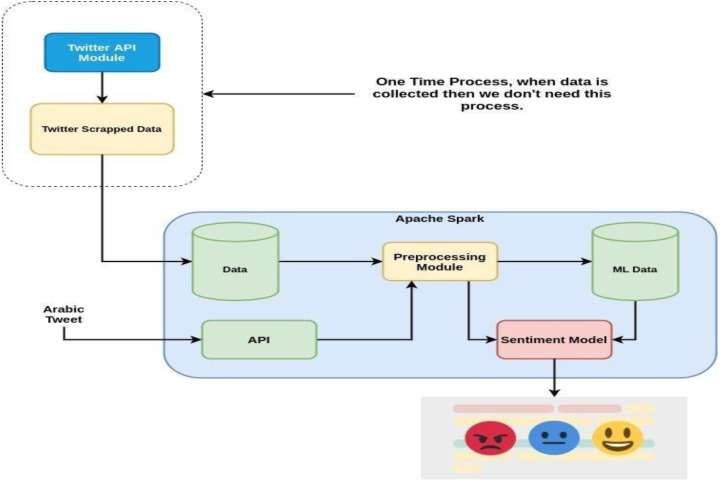
Proposed methodology for the sentiment analysis of Arabic tweets.

The first and most important part of the model is the compilation of a dataset relevant to the e-learning domain. Sentiment analysis is based on the dataset design and few models predict the best decision upon the dataset. There are various techniques for sentiment analysis, including support-vector machine (SVM), decision tree, multi-layer perceptron (MLP) and random forest. A very reliable social media Twitter dataset was selected because Twitter is ideally suited for sentiment analysis. It contains a limit on text length and can easily be scrapped or collected using the Twitter API or third-party libraries. Then a solution is proposed for obtaining distance learning feedback in Saudi Arabia. Figure 1 shows the preliminary steps performed to implement the novel approach.

### Twitter API module for dataset

**Figure 2:**
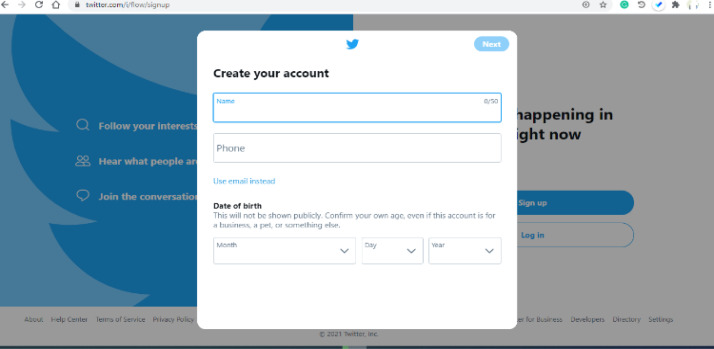
Create your Twitter account.

**Figure 3:**
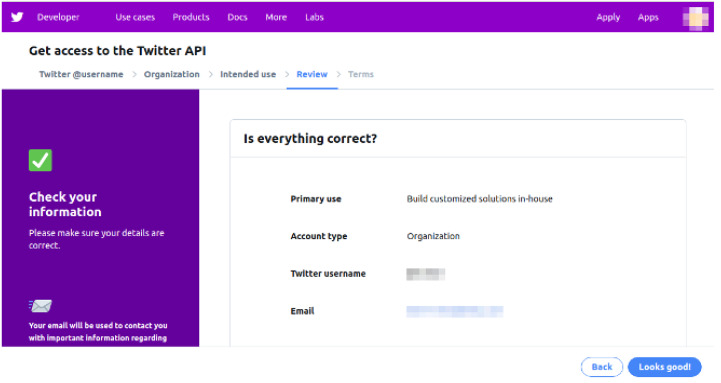
Developer account creation and ‘Get access to the Twitter API’.

This module gets Arabic tweets using Twitter developer credentials and saves the tweets in their raw format. First, we created a Twitter account for our general usage as shown in Fig. 2. After completing this process, a developer account was created as shown in Fig. 3, where an app was created that facilitates scrapping the tweets. In this module, for the first time, a code is written that connects our model with the Twitter API.

For all this work, Twitter developer account credentials were used to scrap the tweets. There is a limit to the number of queries that can be made to get tweets. A paid API was obtained to extract a lot of tweets to be used in our dataset. This process allowed the compilation of a dataset of tweets as large as possible in which the tweet size was generally 512 characters. Then this API extracted around 14,000 tweets, which is enough to build a sentiment dataset for narrow domains. This module is used only once to get the tweets so that we can use this scrapped dataset for training purposes.

### Core elements of a Smart classroom

Preprocessing techniques are applied to clean the tweets, such as removing links, usernames, emojis, and labels. It is very important to create the cleaned dataset (tweets) to get very accurate results from training and testing the model. A bag of words technique was used to remove tweets from the dataset that contained unnecessary information.

The bag-of-words approach simplifies things and is an example from natural language processing (NLP) and information extraction. A text is depicted as a bag of its words in this approach, ignoring syntax and word order while maintaining multiplicity. The proposed model used the most commonly occurring five words to create the bag of words. This bag of words reduced computation and saved time during training. It gives better results when compared with other techniques.

At this stage, data is now in a format that can be used to train a sentiment model. The dataset must be in the form required by the model for sentiment analysis. This data was passed to our model for sentiment analysis. Various algorithms are available that can be used to predict sentiment. After reviewing various algorithms, regression was chosen as it gave us the best results.

### Training model

The scrapped dataset is copied to Apache Spark where parallel computing is used to train spark models. Here, the model was trained on the entire dataset using Apache Spark conventions in which the maximum amount of data can be easily computed. Different packages are required to compute a large amount of data using Python libraries. This is a very important step that is required to manage the scrapped dataset. Regression model was used to predict the sentiment of tweets. There are various other models, including support-vector machines and Naïve Bayes, used for sentiment analysis. The best performing regression model was chosen for the sentiment analysis which performed better and gave the best results, as compared in the result section.

### Flask API for results

Flask API is provided for getting the results of tweets. It can be integrated into websites and smartphone apps for getting the sentiment. In the end, Flask API was used to get the results for our tweets. We pass an Arabic tweet and it gives us a result as to whether it is a positive, negative or neutral tweet.

## Results and Discussion

**Figure 4:**
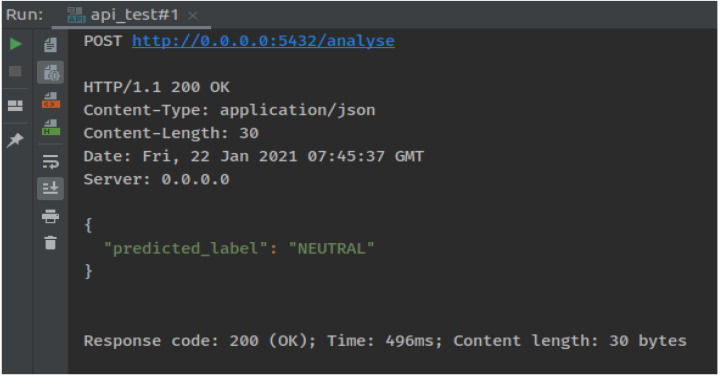
Testing model results.

It was concluded that, proposed model performed best for the Twitter sentiment analysis dataset on the distance learning domain. A large amount of the dataset using Apache Spark was used. The results indicated an accuracy of about 91%, which is the best compared with various other techniques. In the end, an app was developed that receives any tweet from the user and returns whether it is a positive, negative, or neutral tweet. Figure 4 shows the proposed model testing procedure where we pass the test data tweets and the model predicts it to be NEUTRAL. Execution time for each test case is also calculated.

### Comparison of results between different classifiers

The performance of the regression algorithm and SVM algorithm were compared for use in the proposed model. The performance is tested based on the different evaluation measures such as accuracy. There are three types of tweets that are labeled with positive, negative, and neutral. Apache Spark was used to analyze the tweets dataset. Using the logistic regression model achieved a 91% accuracy, whereas using SVM achieved an accuracy of 69%. This indicates that logistic regression performs better than SVM and, as a result, logistic regression was chosed for further evaluation.

Table 1 shows the results for the both Logistic Regression and Support Vector Machine (SVM) algorithm to classify the sentiments on the given dataset. Logistic Regression was selected due to better performance. SVM did not perform well on this data as compared to the Logistic Regression.

### Results from the proposed model

The dataset used for this proposed model was collected in real-time using the Twitter API. After applying all the steps, model was trained to get the best results on the training dataset. The test tweets are unknown to the model and are provided to test the performance of our proposed model. The values that were computed during the model evaluation are shown in Table 2.

Experiments were performed to take different sets of tweets from the dataset. Five thousand tweets were selected from the experiment to train and then tested with the tweets unknown to the model, obtaining accuracy, precision, recall and f1-measure of 82%, 83%, 79% and 81%, respectively. In the second case, 10,000 tweets were taken from the dataset under the same circumstances and the evaluation measures of accuracy, precision, recall and f1-measure obtained are 86%, 85%, 84% and 83% respectively. Finally, 14,000 arabic tweets were taken and applied the same circumstances and obtained accuracy, precision, recall and f1-measure of 91%, 90%, 89% and 90%, respectively. As such, increasing the number of tweets used for training purposes yields better results, as shown in the Table 2.

**Table 1:** Comparison between logistic regression and SVM classifier.

No	Dataset	Classifier name	Accuracy
1	Twitter dataset	SVM	69%
2	Twitter dataset	Logistic regression	91%

**Table 2:** Results evaluated for the proposed model.

No	Dataset	Accuracy	Precision	Recall	F1 Measure
1	5k Tweets Twitter Dataset	82%	83%	79%	81%
2	10k Tweets Twitter Dataset	86%	85%	84%	83%
3	14k Tweets Twitter Dataset	91%	90%	89%	90%

**Figure 5:**
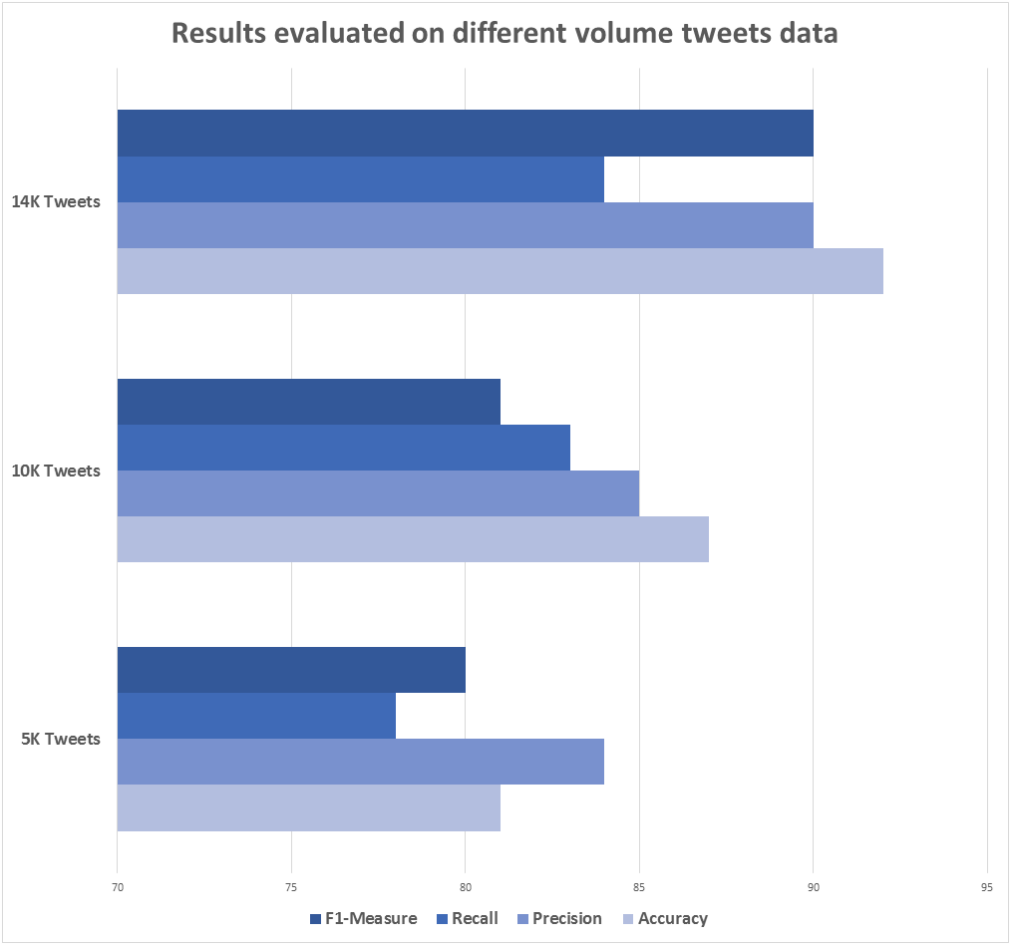
Results evaluation using different data volumes.

Figure 5 shows the evaluated results against different data volumes (size): 5,000, 10,000, and 14,000 tweets. Statistical results are displayed.

### Graphical representation of the results

**Figure 6:**
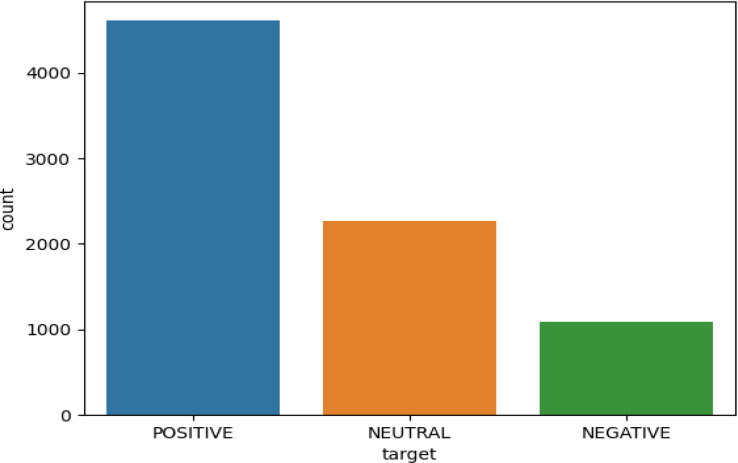
Graphical representation of the people’s behavior.

Applying different approaches to explore different sentiment analysis results on the overall dataset, a graph was plotted that shows the results against the type of tweet: positive, negative, and neutral. The graph in Fig. 6 clearly shows the effect of distance learning on people’s behavior. Finally, applying the technique shows that the majority of people tweeting indicate positive behavior about distance learning in Saudi Arabia. They report on the distance learning policies as they give their feedback using social media (Twitter).

Figure 6 shows the overall visual results for the large volume tweets dataset used for the training and testing of the model. Results indicate more positive sentiment in comparison with the other two classes. This means the positive sentiment analysis class has more impact as compared to other classes.

### Results comparison with state-of-the-art techniques

The proposed model is compared with state-of-the-art methods and the results are better than the existing techniques. The comparison is shown in Table 3. The accuracy value for the proposed model is 91% as compared to the state-of-the-art technique (Aljabri et al., 2021) under the same circumstances. This proves that the proposed model is better and gives outstanding results as compared with the existing techniques.

## Conclusion

Sentiment analysis of real time data is needed specifically for the distance learning domain. People facing issues due to the lack of an optimized system which helps to give the accurate values based on the real time Twitter dataset. The proposed model introduces a method which helps to give a solution for sentiment analysis to check people’s feelings based on Arabic tweets. Machine Learning approaches such as logistic regression and support-vector machine are used for the classification of sentiment. The proposed model performs better than the state-of-the-art. It uses Apache Spark as data storage for the model training. In future, there will be more work by applying deep learning techniques to compute on very large datasets of distance learning Arabic tweets to predict future years. A curious problem faced was using Arabic tweets in Apache Spark on the postman. This problem can be solved by creating an API that can resolve different language syntax problems, as there is no problem with English tweets as they have support from the built-in API.

**Table 3:** Results evaluated for the proposed model.

Dataset	Accuracy	Precision	Recall	F1 Measure
Aljabri et al. (2021)	89%	–	–	–
Proposed Model	91%	90%	89%	90%

##  Supplemental Information

10.7717/peerj-cs.1047/supp-1 Supplemental Information 1 Arabic tweets that have been used

10.7717/peerj-cs.1047/supp-2 Supplemental Information 2 Computer Code

10.7717/peerj-cs.1047/supp-3 Supplemental Information 3 Translated Tweets

## Additional Information and Declarations

### Data Availability

The following information was supplied regarding data availability: The raw data and code are available in the Supplemental Files.
