# Supplementary material for: A machine learning-based approach for sentiment analysis on distance learning from Arabic Tweets
Source: PeerJ Comput Sci. 2022 Jul 26;8:e1047. doi: 10.7717/peerj-cs.1047 (PMC9454973; doi:10.7717/peerj-cs.1047)
Supplement: Supplemental Information 2 [file peerj-cs-08-1047-s002.zip › spark-arabic-sentiment-main/Arabic_Sentiment_Analysis.pdf]

Apache Spark for Arabic Sentiment Analysis

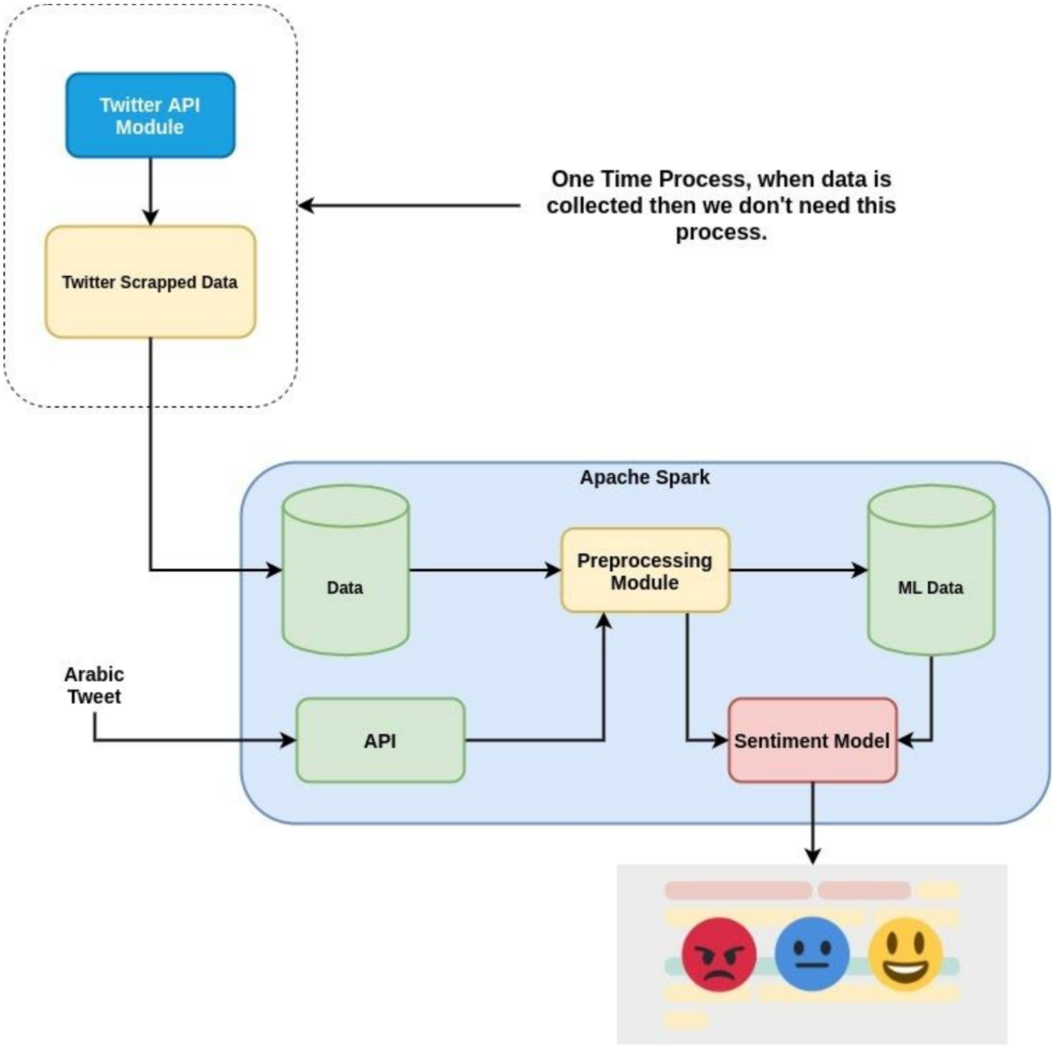

1 - Twitter API Module: This module is used to get Arabick Tweets, it uses Twitter developer credentials and saves the tweets in raw format.

2 - Then this data is copy to apache spark and there it is cleaned using spark conventions of using parallel computing.

3 - Preprocessing is applied to clean the tweet such as removing links, users, emojis, and labels are applied to each tweet for training purposes.

4 - Data is now in a format that can be used to train a sentiment model. I have used the Regression Regression model to predict the sentiment of tweets.

5 - Flask API is provided for getting the results of tweets. It can be integrated into Websites and Smartphone apps for getting the sentiment.
